# Long-term survival achieved by repeated administration of ramucirumab after drug holidays due to proteinuria in recurrent gastric cancer

**DOI:** 10.1186/s40792-021-01185-9

**Published:** 2021-04-20

**Authors:** Naoyuki Uehata, Keita Kouzu, Hironori Tsujimoto, Hidekazu Sugasawa, Kotaro Wakamatsu, Yoji Kishi, Hideki Ueno

**Affiliations:** Department of Surgery, National Defense Medical College, 3-2 Namiki, Tokorozawa, Saitama 359-8513 Japan

**Keywords:** Advanced gastric cancer, Lymph node recurrence, Ramucirumab, Chemotherapy, Re-shrank, Proteinuria

## Abstract

**Background:**

The prognosis of recurrent and unresectable gastric cancer remains poor despite the development of multidisciplinary treatments. Ramucirumab (RAM) has been proven effective against unresectable or recurrent gastric cancer. However, its administration is often discontinued because of adverse events, including hypertension and proteinuria. We report a patient with recurrent gastric cancer involving the paraaortic lymph node (PALN), who achieved long-term survival after repeated RAM administration following long-term drug holidays due to proteinuria.

**Case presentation:**

A 79-year-old woman was diagnosed with advanced gastric cancer (cT4aN2) with PALN metastasis. Seven courses of S-1 plus cisplatin (SP) achieved downstaging. A distal gastrectomy with D2 lymphadenectomy was performed as a conversion surgery. The pathological diagnosis was ypT3N2M0. The dissected PALN did not contain viable cancer cells. CT and positron emission tomography/CT scans revealed PALN recurrence 1 year after the surgery. S-1 plus oxaliplatin (SOX) therapy was initiated. The recurrent PALN enlarged after seven courses of SOX therapy. Paclitaxel (PTX) plus ramucirumab (RAM) therapy was initiated as second-line chemotherapy. After three courses of PTX plus RAM therapy, a partial response was observed. PTX was discontinued because of a hematological adverse event 3.5 months after PALN recurrence. Disease progression was not observed after six courses of RAM monotherapy. However, RAM caused proteinuria and was withdrawn for 7 weeks. The recurrent PALN was enlarged on CT, and RAM monotherapy was resumed at a reduced dose of 6 mg/kg. The lesion subsequently shrank, but 4 + proteinuria occurred after three courses of RAM monotherapy. Thus, RAM was discontinued. The patient had chemotherapy-free days for 14 months until the PALN was re-enlarged to 13 mm in size. The three administrations of RAM successfully controlled PALN metastasis and proteinuria for 3 years.

**Conclusion:**

In conclusion, even if RAM withdrawal led to disease progression, re-administration of RAM monotherapy while considering its side effects reduced the tumor size and provided long-term survival benefits.

## Background

Gastric cancer is one of the most common cancers worldwide, and it was the third leading cause of cancer-related deaths in 2018 [1]. In Japan, S-1 plus cisplatin has been recommended as the first-line regimen based on the results of two phase III trials (SPIRITS trial and JCOG 9912 trial) by Japanese gastric cancer treatment guidelines [2]. Following the results of the RAINBOW trial, paclitaxel and ramucirumab (RAM) became the new standard of care as the second-line regimen [2].

According to the RAINBOW and REGARD trial results, RAM has been widely recognized as one of the standard chemotherapy drugs for unresectable advanced or recurrent gastric cancer [2]. However, the RAM-based adverse events, such as severe hypertension, thromboembolism, and proteinuria, make continuing treatment difficult.

We report a case of recurrent gastric cancer who achieved long-term survival after repeated administrations of RAM following drug holidays due to proteinuria development.

## Case presentation

**Fig. 1:**
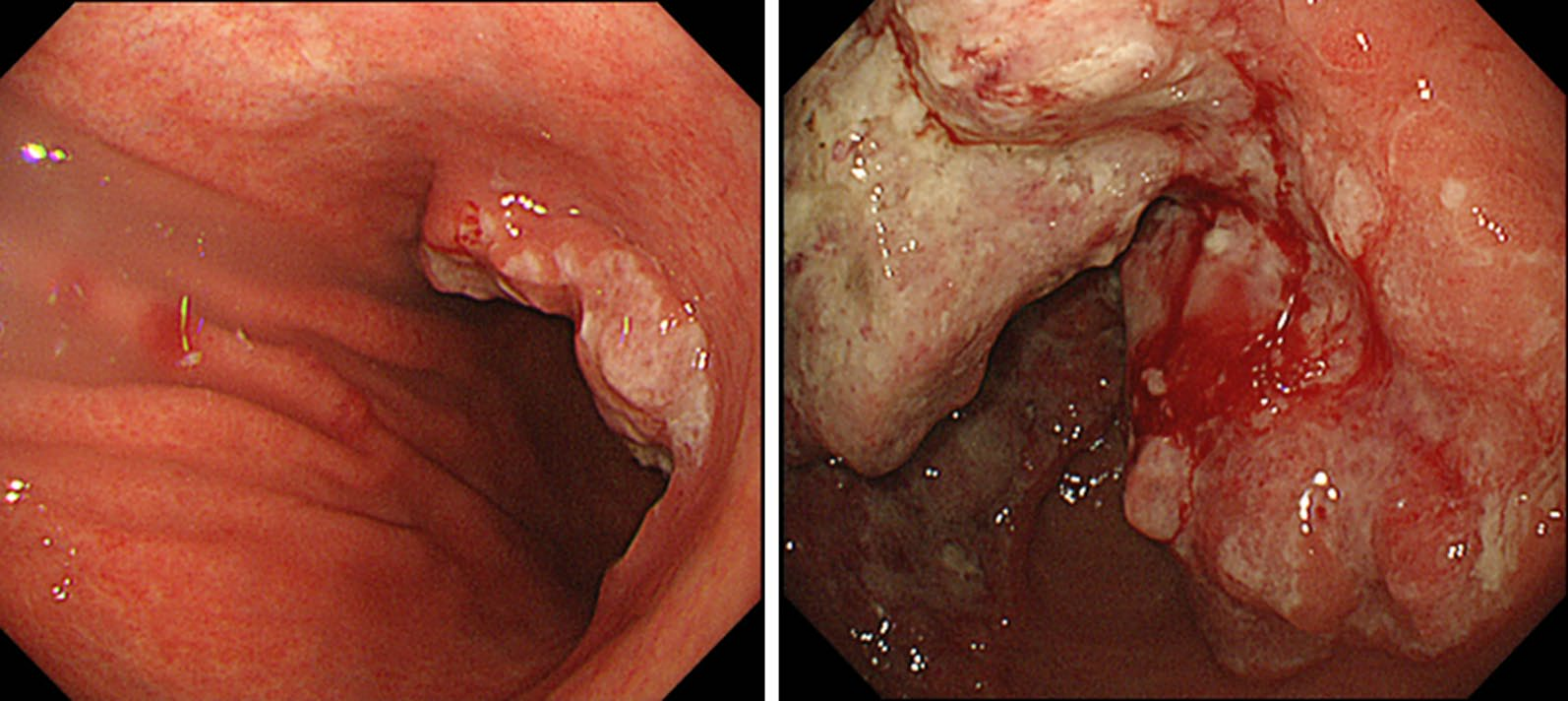
Gastrointestinal endoscopy at the time of the initial examination revealed a Type 3 tumor, which mainly occupied the region from the lower gastric body to gastric antrum

**Fig. 2:**
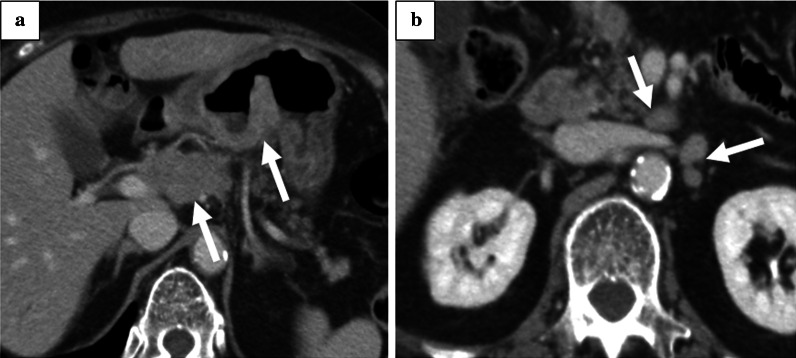
Contrast-enhanced CT at the time of diagnosis. **a** Thicker gastric wall and enlarged regional lymph nodes (No. 8a). **b** High-density and enlarged No. 16a2 lymph nodes

A 79-year-old woman with hypertension and type 2 diabetes was referred to our hospital for gastric cancer. Gastrointestinal endoscopy revealed a type 3 tumor located in the lower gastric body (Fig. 1). Laboratory tests revealed slight anemia and elevated tumor markers (Table 1). Histopathological examination of a biopsy specimen revealed moderately differentiated tubular carcinoma and weak (1 +) HER2 positivity on immunohistochemical staining. Contrast-enhanced computed tomography (CT) showed a thickened gastric wall and swollen regional (No. 6 and 8a, Fig. 2a) and para-aortic lymph nodes (LNs) (No. 16a2 LNs, Fig. 2b). The patient was diagnosed with advanced gastric cancer (cT4aN2 [No. 3a, 6, 8a] M1 [LYM], stage IV), according to the 8th edition of the Union for International Cancer Control TNM Classification of Malignant Tumors [3].

**Table 1:** Laboratory data at the patient's first visit

*Biochemistry*		*Peripheral blood*	
TP (g/dL)	6.6	WBC (/μL)	*11,200*
Alb (g/dL)	*3.6*	RBC (× 10^6^/μL)	4.01
BUN (mg/dL)	13	Hb (g/dL)	11
Cre (mg/dL)	0.63	Plt (× 10^4^/μL)	49.2
Na^+^ (mmol/L)	139		
K^+^ (mmol/L)	4.4	*Tumor marker*	
Cl ^−^(mmol/L)	105	CEA (ng/mL)	3.4
CRP (ng/mL)	*1.2*	CA19-9 (U/mL)	*49.0*

Italic values mean that they are higher than the normal range.

*TP* total protein, *Alb* albumin, *BUN* blood urea nitrogen, *Cre* creatinine, *CPR* C-reactive protein, *WBC* white blood cell, *RBC* red blood cell, *Hb* hemoglobin, *Plt* platelet, *CEA* carcinoembryonic antigen, *CA* carbohydrate antigen

S-1 plus cisplatin (SP) therapy (S-1 80 mg/m^2^, cisplatin 60 mg/m^2^) was initiated. After seven courses of SP therapy, CT revealed significantly shrunken LNs, but the primary lesion remained unchanged on gastrointestinal endoscopy. Serum tumor marker studies showed elevated level of carbohydrate antigen 19-9 (CA19-9, 84.1 U/mL) and normal level of carcinoembryonic antigen (CEA, 4.8 ng/mL).

Since laparoscopic exploration revealed no unresectable factors, such as peritoneal metastasis and positive cytology, the patient underwent distal gastrectomy with D2 LN dissection and picking of Nos. 16a2 and 16b1 as conversion surgery with curative intent. The patient was discharged 11 days postoperatively, with an uneventful postoperative course.

The resected specimen revealed a type 3 tumor in the lower gastric body. Pathological examination revealed tumor involvement in the subserosa layer. Four out of 57 dissected LNs were positive for metastasis, and the dissected PALN did not contain viable cancer cells. The final diagnosis of the cancer was ypT3N2M0 stage IIIA. Adjuvant chemotherapy was not administered because of patient refusal. Serum CA19-9 levels decreased to 40.1 U/mL and CEA levels unchanged at 4.9 ng/mL after surgery.

**Fig. 3:**
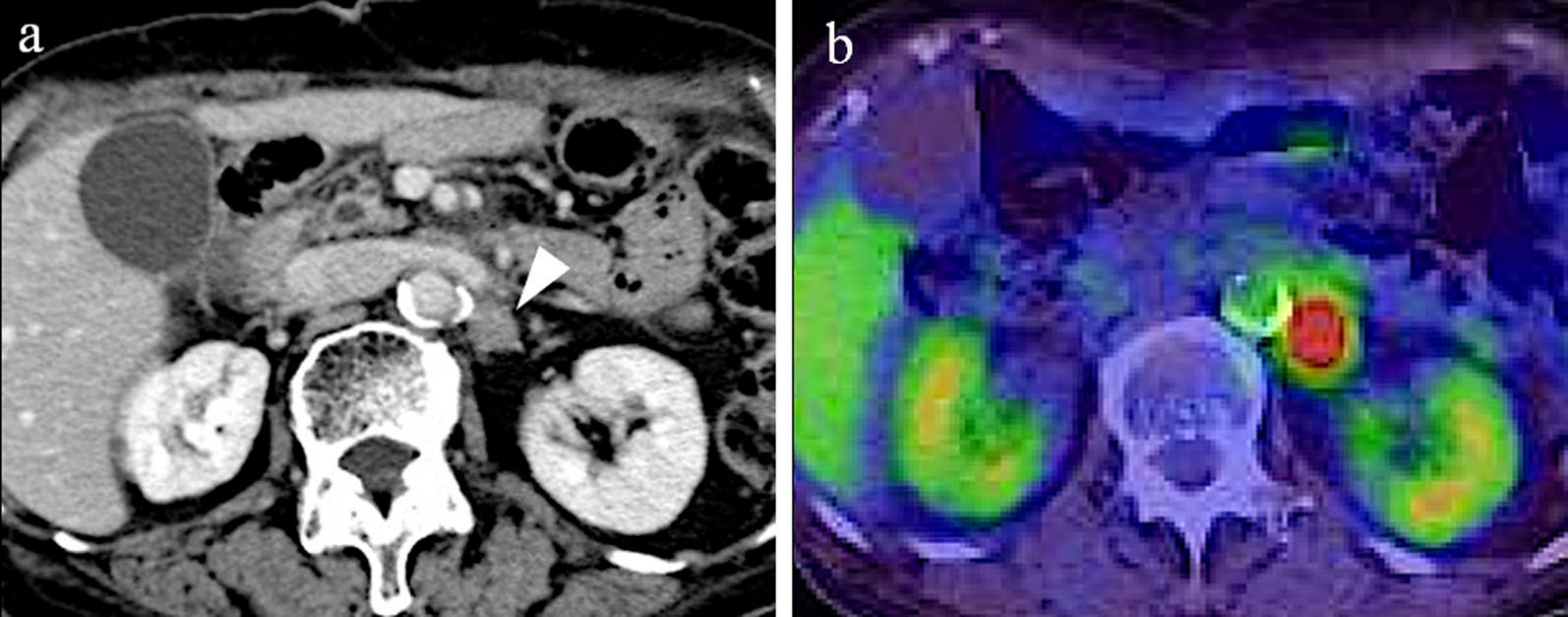
**a** Contrast-enhanced CT and **b** fluorodeoxyglucose-positron emission tomography/CT at recurrence of cancer appeared in No. 16b1. The size was 14.6 mm

**Fig. 4:**
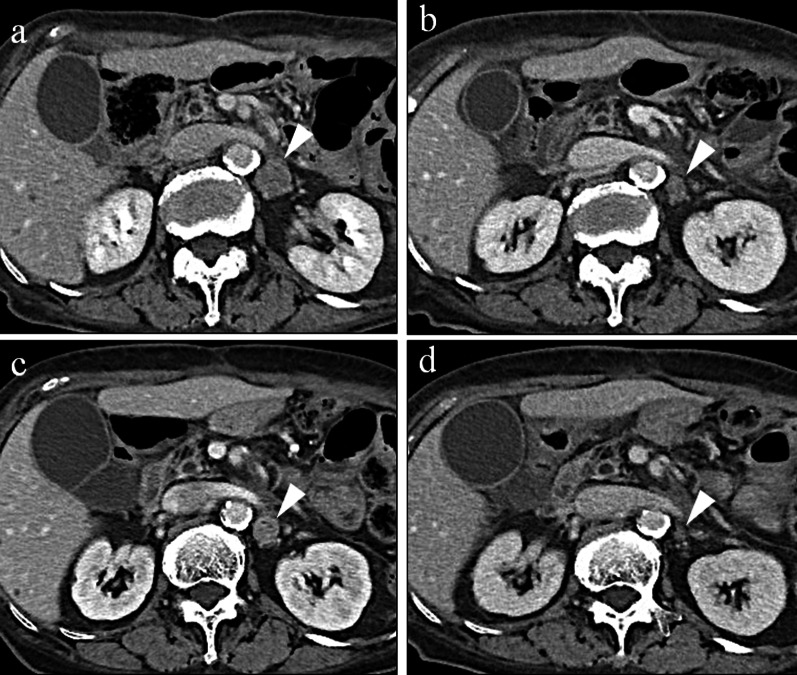
**a** Recurrent No. 16b1 enlarged to 20.1 mm after SOX therapy. **b** Lesion shrank to 15.6 mm after three courses of RAM + PTX therapy. **c** Lesion enlarged to 18.1 mm during the first RAM withdrawal period. **d** Lesion again shrank to 10.5 mm with resuming RAM administration.

**Fig. 5:**
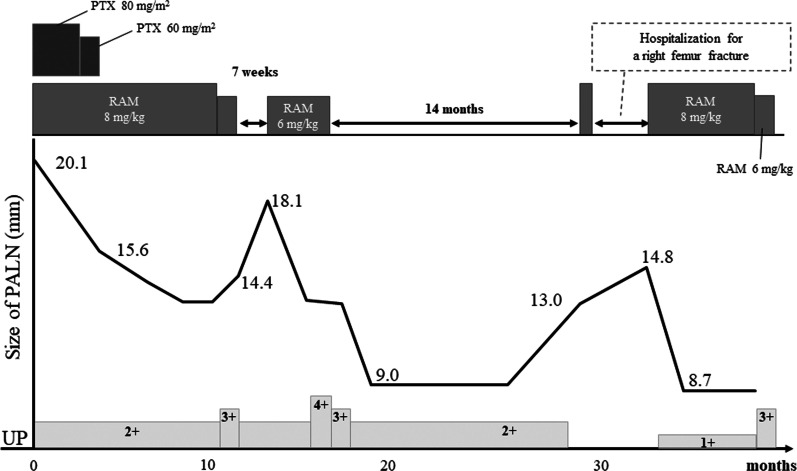
Timeline of this patient after the initiation of second-line chemotherapy with PTX plus RAM. Although the recurrent No. 16b1 lymph node grew during RAM's withdrawal period, it shrank again after resumed RAM

One year after gastrectomy, a CT scan revealed swelling of the paraaortic (No. 16b1 LN) (Fig. 3a) and left supraclavicular LNs. In addition, CA19-9 remained high at 40.1 U/mL, CEA elevated to 6.1 ng/mL, and a positron emission tomography/CT scan showed abnormal accumulation of fluorodeoxyglucose in No. 16b1 LN (Fig. 3b). For the recurrent gastric cancer, S-1 plus oxaliplatin (SOX) therapy (S-1 80 mg/m^2^/day, oxaliplatin 100 mg/m^2^) was initiated as the first-line chemotherapy. After the seventh course of SOX therapy, the supraclavicular LN disappeared, but the paraaortic LN became enlarged (Fig. 4a). Therefore, PTX plus RAM therapy (PTX 80 mg/m^2^ and RAM 8 mg/kg) was initiated as the second-line regimen (Fig. 5). After three courses of PTX plus RAM therapy, CT revealed shrunken paraaortic LN (Fig. 4b), and PTX caused severe adverse effects, including grade 3 neutropenia and grade 2 peripheral neuropathy. The dose of PTX was reduced to 60 mg/m^2^ in the third course, and PTX was discontinued from the fourth course. CEA elevated to 11.2 ng/mL, but the size of paraaortic LN gradually shrank and CA19-9 decreased to 18.6 U/mL at the fifth course of RAM monotherapy. However, 3 + proteinuria and hypertension occurred before the sixth course of RAM monotherapy. The RAM was reduced to 6 mg/kg, and the patient observed a drug withdrawal period of 7 weeks. Because the size of paraaortic LN was enlarged after withdrawal period (Fig. 4c), 6 mg/kg of RAM monotherapy was resumed. After resuming RAM administration, the paraaortic LN significantly shrank again (Fig. 4d). However, 4 + proteinuria occurred and the urine protein–creatinine ratio was 5.29. Thus, RAM was withdrawn again, and regular CT and urinalysis were scheduled with sufficient informed consent. During the 14-month withdrawal period, hypertension was controlled by a calcium blocker, proteinuria remained at 2 + , and LN enlargement was not observed. Since the paraaortic LN was enlarged to 14 mm, RAM (8 mg/kg) was administered again. Unfortunately, the patient had a femoral fracture shortly after resumption and needed further drug withdrawal. During this period, the paraaortic LN grew to 14.8 mm. After the patient recovered, RAM was readministered, and the paraaortic LN shrank to 8.7 mm again. RAM monotherapy was continued while carefully observing adverse events, such as proteinuria and hypertension, 57 months after the conversion surgery.

## Discussion

RAM is a molecular target drug classified as an angiogenesis inhibitor and a fully human IgG1 monoclonal antibody, targeting vascular endothelial growth factor (VEGF) receptor-2 (VEGFR-2) [4, 5]. RAM prevents the binding between VEGF and VEGFR-2, and reduces tumor growth by preventing tumor angiogenesis [6]. The benefits of RAM, for advanced gastric or gastroesophageal junction adenocarcinoma, were confirmed by the REGARD and RAINBOW trials. The REGARD trial showed more significant prolongation of overall and progression-free survival in the RAM group than in the placebo group, with a median duration of disease control of 4.2 months and a median survival time of 5.2 months in the RAM group [5]. The RAINBOW trial also showed that both overall and progression-free survival were longer in the PTX + RAM group than in the placebo + PTX group [4]. In the present case, PTX + RAM therapy was initiated as second-line chemotherapy for recurrent paraaortic LNs. However, the hematological toxicity caused by PTX led to a shift to RAM monotherapy. Our patient continued RAM monotherapy and achieved disease control for more than 36 months after the initiation of RAM monotherapy with two drug holidays. This period was 31 months longer than the median duration of disease control in the REGARD trial.

The incidences of grade 3 or higher adverse events in the REGARD and RAINBOW trials were 56.8% and 81.7%, respectively [4, 5]. The RAM-specific side effects included hypertension (16% and 25%), bleeding or hemorrhage (13% and 42%), and proteinuria (3% and 17%) for the REGARD and RAINBOW trials, respectively. In addition, neutropenia (54.4%) and neuropathy (45.9%) caused by PTX were reported as side effects of PTX + RAM therapy in the RAINBOW trial.

Proteinuria is a common adverse event associated with RAM, and severe proteinuria requires therapy cessation in responsive patients [7]. RAM-induced proteinuria results from a renal-glomerular disorder caused by preventing interaction between VEGF, produced by podocytes, and VEGFR-2, expressed by glomerular endothelial cells [8, 9]. Proteinuria is a risk factor for cardiovascular and renal disease. Regular qualitative urine protein testing is recommended during RAM treatment to monitor proteinuria's appearance, based on the results of the REGARD and RAINBOW trials [4, 5]. Withdrawal and dose reduction are recommended in the case of proteinuria of 2 g/day or more but less than 3 g/day during treatment with RAM, and discontinuation is recommended in the case of proteinuria of 3 g/day or more or nephrotic syndrome. When proteinuria 4 + was observed, it is common to discontinue RAM and shift to third-line therapy. However, we chose to withdraw RAM again and follow-up in this case because of the patient's request. After the withdrawal period, we resumed RAM, because RAM remained to have the high therapeutic efficacy for this patient while no adverse events other than proteinuria.

To our knowledge, there is no information on the effectiveness of resuming RAM or other angiogenesis inhibitors, such as bevacizumab, in responsive patients who stopped receiving angiogenesis inhibitors due to severe adverse events. In colon cancer, oxaliplatin is discontinued and readministered in the FOLFOX regimen to reduce neurotoxicity [10]. Thus, it may be possible to apply the stop-and-go policy while monitoring proteinuria during RAM monotherapy.

## Conclusions

We encountered a rare case of long-term survival using RAM as the second-line chemotherapy for the recurrence of paraaortic LNs. This was a valuable case, wherein the recurrent gastric cancer, which progressed during the RAM withdrawal period, shrank again upon resuming RAM administration, resulting in long-term survival. In gastric cancer patients who are responsive to RAM, re-administration of RAM may provide a significant objective response. Even after withdrawing RAM treatment due to adverse effects, RAM is still a viable therapeutic option in responsive patients.

## Data Availability

All the data in this manuscript are available within the manuscript.

## Abbreviations

CT: Computed tomography
LN: Lymph node
PD: Progressive disease
PR: Partial response
PTX: Paclitaxel
RAM: Ramucirumab
SD: Stable disease
SOX: S-1 plus oxaliplatin
SP: S-1 plus cisplatin
VEGF: Vascular endothelial growth factor
VEGFR-2: Vascular endothelial growth factor receptor-2
